# Feasibility of a Responsibility-Based Leadership Training Program for Novice Physical Activity Instructors

**DOI:** 10.3389/fpsyg.2021.648235

**Published:** 2021-08-06

**Authors:** Hanna-Mari Toivonen, Mary Hassandra, Paul M. Wright, Martin S. Hagger, Nelli Hankonen, Kaarlo Laine, Taru Lintunen

**Affiliations:** ^1^Faculty of Sport and Health Sciences, University of Jyväskylä, Jyväskylä, Finland; ^2^School of Physical Education, Sport Sciences, and Dietetics, University of Thessaly, Volos, Greece; ^3^Department of Kinesiology and Physical Education, Northern Illinois University, DeKalb, IL, United States; ^4^Psychological Sciences, University of California, Merced, CA, United States; ^5^Faculty of Social Sciences, University of Helsinki, Helsinki, Finland; ^6^LIKES Research Centre for Physical Activity and Health, Jyväskylä, Finland

**Keywords:** shared leadership, positive youth development, feasibility, teaching personal and social responsibility model, novice instructor, leadership training, physical activity

## Abstract

Most coaches and instructors would like to teach more than just sport skills to their athletes and children. However, to promote athletes’ or children’s holistic development and teach them to take responsibility and lead, requires the coaches and instructors to first master the skills themselves. Therefore, feasible, high quality leadership training programs where coaches and physical activity instructors are taught to teach and share leadership are needed. The aim of the current study was to evaluate the feasibility of a leadership training program to optimize it and to determine whether to proceed with its evaluation. In the leadership training program, eight Finnish novice physical activity instructors, aged 18 to 22, were taught to promote positive youth development, personal and social responsibility, and shared leadership in a physical activity context. The participants had minimal to no leadership training or experience. The training program consisted of seven meetings totaling 20 h. Helllison’s teaching personal and social responsibility (TPSR) model was the theoretical and practical framework of the training program. Feasibility of the leadership training program was evaluated across four domains of an evidence-based framework: demand, practicality, acceptability, and implementation fidelity. Data of the current complex intervention were collected with application videos, questionnaires, researcher’s log, lesson plans, video recordings, and a semi-structured focus group interview. The quantitative data were analyzed using descriptive statistics and the qualitative data using deductive and inductive content analysis. There was a demand for the leadership training program. The training program was perceived as practical and highly acceptable by the novice instructors and the trainers, and implemented with fidelity, indicating high overall feasibility. No implementation issues were found. Consequently, the current leadership training program has a high probability of efficacy and can be accepted for further evaluation.

## Introduction

Group-based physical activity sessions offer plenty of opportunities to promote positive youth development (PYD). PYD is a strength-based process seeking to engage youth in activities that nurture a wide range of developmental assets (i.e., life skills) and support young people to grow into happy, healthy, productive, and contributing members of society (Catalano et al., 2004). Although psychosocial development is an important objective of young people’s physical activity programs, it is often not accomplished (Côté et al., 2008). Sport participation alone does not guarantee the development of life skills, such as leadership, but these skills need to be taught proactively (Gould and Voelker, 2010; Lintunen and Gould, 2014).

Shared leadership is a group-centered approach to leadership characterized by collective lateral interaction stemming from all or most group members and distributed widely across group members (Zhu et al., 2018). Vertical leadership is a person-centered approach to leadership (i.e., formal leader). Both structures of group leadership are needed as they supplement each other (Carson et al., 2007; Fausing et al., 2015). Formal leaders can initiate, facilitate, and maintain shared leadership in the group (Pearce, 2004). Chiu et al. (2016) found that in teams where shared leadership was used, the formal leaders displayed humility (e.g., by admitting to their own limitations) and allowed team members to take responsibility, which led to the team members embracing shared leadership. In line with their findings, Fransen et al. (2020) found that best coaches adopted a shared leadership approach. Empowering the players strengthened the leadership quality of the players and enhanced the players’ perception of the coach as a good leader. Furthermore, Zhu et al. (2018) found that there have been different approaches to “what is being shared in shared leadership?” and “what is the process through which leadership is shared?” In shared leadership, a specific leadership style or the overall leadership can be shared among the members of the group. The sharing of leadership can happen overtime, can be done as a group, or can be done by taking turns or dividing roles. Whichever the case, shared leadership requires more than just a decision to share leadership, and it requires leadership skills from the formal leader and all members of the group. Therefore, coaches and physical activity instructors should be trained to understand and share leadership. In the current study, the shared leadership was embedded in the training program. Novice instructors were, for example, given peer coaching roles, leadership roles in planning, and youth instructor roles. The novice instructors’ leadership and responsibility were gradually increased throughout the training program.

Hellison’s (1985, 2011) teaching personal and social responsibility (TPSR) model is one of the most comprehensive frameworks frequently used to promote PYD and shared leadership. The model was originally developed for underserved children to empower them and gradually teach them to become responsible leaders. It has since been used with various populations and contexts around the globe (e.g., Rantala and Heikinaro-Johansson, 2007; Hassandra and Goudas, 2010; Beaudoin, 2012; Gordon, 2012; Jung and Wright, 2012), including afterschool physical education context (Gordon et al., 2016). The model uses physical activity as a vehicle for teaching values and life skills, such as autonomy, goal setting, leadership, and teamwork. The main goals of the model are to promote personal (i.e., self-regulation and effort) and social (i.e., cooperation and leadership) responsibility and to apply the responsibility skills in other settings, such as school, sports, community, or home. TPSR has been shown to be a useful model for teaching life skills in numerous youth intervention programs resulting in a range of positive behavioral, social, emotional, psychological, and educational outcomes (Hellison and Walsh, 2002; Pozo et al., 2018).

TPSR approaches leadership from the responsibility perspective (Martinek and Hellison, 2009). Giving instructors a large amount of autonomy and responsibility too early in their training, and without providing them with sufficient instruction on how to utilize it effectively, may negatively impact their ability to cope with their role and may also undermine their confidence. Therefore, responsibility and leadership should be shared to the instructors when they are ready for it and to the extent, they are ready. In TPSR-based programs, sharing is about giving meaningful and genuine voices and choices to everyone in the group. The goal is for everyone in the group to learn to take personal and social responsibility. Personally, a leader needs to learn the importance of effort and learn to set and work toward personal goals. Socially, a leader needs to develop their relational skills and values, be able to share their perceptions and ideas to enhance the group and to ensure that everyone feels safe and heard in the group. Therefore, TPSR practices are based on five levels of responsibility, which are utilized when TPSR-based leadership training is given to young people. The five levels of responsibility are as follows: (1) respect for the rights and feelings of others (2) effort/participation (3) self-direction (4) helping others/leadership, and (5) transferring responsibility to other contexts. These levels of responsibility cannot be learned in a short period of a time but should be practiced step-by-step over an extended period.

When the basics of being responsible have been learned, leadership can be further developed by providing opportunities to help, teach, or coach peers (Martinek and Hellison, 2009). This requires guidance and support as well as feedback and reflection. For example, in a recent project in Belize eight of the 36 coaches were elected by the group to be the leadership team to guide and support their peers and the direction of their project (Wright et al., 2016). Providing opportunities for peer leadership will also create opportunities to learn to follow others’ lead and lead together. Peer leadership is one form of shared leadership. Once peer leadership has become a norm in a group, more responsibility and more challenging leadership opportunities can be provided. For example, Cutforth and Puckett (1999) in their apprentice teacher program empowered young people to work in pairs as a leadership team working with younger children. However, it is important to ensure that support is available to help to overcome frustration and challenges the leadership roles entail. Especially, with young people who do not have much experience in leadership, they can find the amount of responsibility daunting even if they are not leading the group alone. When they are ready for even more extended experiences, the transfer of leadership from one context to another can be emphasized. Gordon et al. (2016) had a feature in their afterschool youth program “Project Leadership” that involved students not just taking on peer coaching roles in the program but taking on a leadership role in planning and hosting a school-wide wellness night as a service project for the whole school community. Through the extended leadership experiences, the trained people can eventually become leaders in their communities and in society. They can also take formal leadership roles and promote and teach shared leadership. Specific strategies to help participants to practice the levels of responsibility in the TPSR model are relational time, awareness talk, physical activity with embedded life skills, group meeting, and self-reflection.

According to Hellison (2011), TPSR program leaders need to create positive relationships with the participants, gradually empower them by sharing responsibility and leadership with them, integrate responsibility roles and concepts into physical activity, and address transfer of life skills from physical activity context to other settings. Leadership skills should be developed in young people to achieve success in their lives and to display positive youth behaviors. Even experienced teachers, coaches, or instructors who recognize the importance of teaching life skills often lack confidence and toolset to effectively teach them (Koh et al., 2017). Therefore, a TPSR-based leadership training program could help teachers, coaches, or instructors to gain knowledge and tools to promote PYD, responsibility, and shared leadership. Among young novice instructors, the need is even greater. Rather than learning leadership solely through trial and error or by observing others, novice instructors would benefit from having a framework and guidelines to follow. The novice instructors could be encouraged to adopt a shared leadership approach from the start and taught to identify the strengths of the participants and include the participants in decision making (Fransen et al., 2020).

There is a lack of research on developing young adults to become leaders, although early adulthood is an especially important time for leadership growth (Karagianni and Montgomery, 2018). Training physical activity program leaders is a key factor in developing quality PYD programs (Martinek and Hellison, 2009). Although many TPSR-based leadership trainings exist (e.g., Escartí et al., 2010; Romar et al., 2015; Wright et al., 2016; Alcalá et al., 2019), to ensure quality implementation and to enhance the ability to interpret findings and generate theory, comprehensive, systematic, and rigorous evaluations of the leadership training programs need to be performed (Wright et al., 2018).

Feasibility studies examine whether the planned intervention and evaluation can be performed (O’Cathain et al., 2015) and whether the intervention approach should be accepted or discarded, so that only those interventions that are worth testing (i.e., have a high probability of efficacy) are advanced (Bowen et al., 2009). Feasibility studies aid researchers in identifying potential implementation issues (Taylor et al., 2006) and examining key uncertainties (Craig et al., 2008), to determine what needs modification and how changes might occur (Bowen et al., 2009). Therefore, feasibility testing should take place prior to evaluation of the effectiveness or dissemination of the program (O’Cathain et al., 2015). In feasibility testing, mixed methods might yield more conclusive and innovative results than qualitative or quantitative methods alone (Bowen et al., 2009).

Several TPSR studies have examined one aspect of feasibility, namely, the implementation fidelity (Pascual et al., 2011; Lee and Choi, 2015; Cryan and Martinek, 2017; Richards and Gordon, 2017; Escartí et al., 2018). Implementation fidelity refers to the degree to which a program is implemented as intended by the program developers (Breitenstein et al., 2010). Previous TPSR-based studies have been examining fidelity of the teachers’ implementation of the responsibility levels, the TPSR-based teaching strategies, the daily lesson format, and the TPSR themes, as well as the prevalence of responsible student behaviors (Pascual et al., 2011; Lee and Choi, 2015; Cryan and Martinek, 2017; Richards and Gordon, 2017; Escartí et al., 2018). In most of these studies, the fidelity was only moderate and there were significant differences in the fidelity depending on the program leader (Pascual et al., 2011; Richards and Gordon, 2017; Escartí et al., 2018). On the other hand, TPSR-based professional development programs, typically targeting physical education teachers, were found to improve fidelity of the programs (Hemphill et al., 2015; Lee and Choi, 2015), which suggests that continuous training of program leaders is an important part of implementation fidelity of the program. However, high implementation fidelity does not alone mean that the program should be implemented. In some studies (e.g., Wright et al., 2018), also acceptability in terms of instructor satisfaction of the training has been considered. In addition to implementation fidelity and acceptability, other domains of feasibility: demand, practicality, adaptation, expansion, integration, and limited efficacy (Bowen et al., 2009) of the program can be evaluated to ensure sustainability, affordability, and the likelihood of successfully implementing the program in the future (Shields et al., 2018).

The aim of the current study is to assess the feasibility of a 20-h TPSR-based leadership training program for novice physical activity instructors. The TPSR-based leadership training program is presented in detail in the protocol article of Toivonen et al. (2021). The protocol article describes the development of a TPSR-based leadership training program, the content, and a plan for an intervention study in which novice instructors learn to understand and apply the TPSR model in practice. Assessing the feasibility of the leadership training program is important in order to optimize the intervention and to determine whether the program should be accepted for further evaluation. In the current study, the feasibility of the leadership training program is evaluated across four of the domains of an evidence-based framework for feasibility studies: demand, practicality, acceptability, and implementation fidelity (Bowen et al., 2009). The implementation fidelity is further evaluated in terms of four dimensions: adherence, dose, quality of delivery, and program differentiation (Dane and Schneider, 1998).

## Materials and Methods

### Participants and Setting

Recruitment of the novice instructors occurred at four local high schools and one vocational school. An invitation message was sent to about 7,000 current students through online message boards and thousands of former students through alumni mailing lists. Specific numbers are unknown because the invitation was circulated by the schools themselves. The applicants had to be adults (18 years or older) with some experience in organized physical activity, for example, as a participant. However, they could not have extensive coaching, teaching, or instructing experience (full-time position for over 6 months or part-time position for over 1 year) or training (high-level coaching or teaching certificate, or multiple smaller trainings). They had to be interested in physical activity and in helping youth with physical activity and beyond. They also had to be willing to participate in a 20-h leadership training program during summertime. After expressing the initial interest to participate, applicants were asked to send an application video containing information on their age, gender, education, occupation, physical activity background, favorite sports, instructing experience, instructing training, and motivation to participate in the training program and work with young people. Covariate adaptive randomization (Treasure and MacRae, 1998; Lin et al., 2015) was used to select eight novice instructors (4 females and 4 males) aged 18 to 22, to participated in the TPSR-based leadership training program. The randomization and selection processes were performed as described in the study protocol (Toivonen et al., 2021).

All eight participants were Caucasian and fluent in Finnish. Five were starting their final year in high school, one had completed vocational education, and two were starting studies in a University of Applied Sciences. The novice instructors had backgrounds in a variety of sports, such as basketball, mogul skiing, circus, aikido, floorball, and boxing, and five of them had played soccer. Six reported team sports, one combat sports, and one outdoor physical activity as their favorite sport. Six of the novice instructors did not have any sort of previous leadership training, whereas two had completed a short confirmation camp counselor training organized by a church and had been counselors at a confirmation camp. The novice instructors also did not have much experience in leading a group. Two of the novice instructors had been tutors at school, and four had been assisting in a sport camp, a sport tournament, or an afterschool program. One had coached children for one winter, and one had no formal leadership experience. All novice instructors are referred to with pseudonyms.

The leadership training program was organized in a city with a population of approximately 140,000 in Central Finland. The training program was implemented by the first and the last author in a university classroom and gymnasium as well as different sport facilities around the city. Three video cameras were used to record the training. In the classroom, a projector and a screen were used. In the gymnasium and other sport facilities, a variety of physical activity equipment were utilized. Snacks were provided during the first five meetings. This study was carried out in accordance with “Responsible conduct of research and procedures for handling allegations of misconduct in Finland” – guidelines by the Finnish Advisory Board of Research Integrity. All participants gave written informed consent. The Ethics Committee of the University of Jyväskylä (No. 29062015) approved the study.

### Leadership Training Program

The leadership training was an intensive 20-h program consisting of seven meetings organized over five weeks. The theoretical and practical framework is the TPSR model (Hellison, 1985, 2011). Research has shown that coaches learn best through a combination of non-formal (e.g., workshops) and intentional or incidental informal (e.g., observing other coaches) experiential opportunities, and formal lecture-based courses, accompanied with reflective process (Jarvis, 2006; Cushion et al., 2010). The leadership training program combined these different learning situations as an attempt to optimize learning. Consequently, five core components of the leadership training program were theory, activity, experiential learning, evaluation, and experiences of leadership.

The first meeting of the novice physical activity instructors’ leadership training program consisted of practical issues and getting to know one another. The second meeting included a lecture on the TPSR model along with activities to create a safe, trustful, supportive, and positive learning environment. The third meeting had two model lessons, which demonstrated how to implement the TPSR model in physical activity instruction and provided the novice instructors experiences of being a participant in TPSR-based physical activity lessons. The fourth meeting consisted of the first set of practice teaching lessons organized for the peer novice instructors in pairs, which provided the participants their first leadership experience. The fifth meeting included the second set of practice teaching lessons delivered for young volunteer athletes. The sixth and seventh meetings were organized separately for each pair. The sixth meeting consisted of an observation of a sport practice and providing feedback for the coaches on their leadership behaviors based on the observation. The seventh meetings included the third set of practice teaching lessons organized for a sport team or sport group. A more detailed description of the novice instructors’ leadership training program, the included activities, and information needed to replicate the program can be found from the protocol article (Toivonen et al., 2021).

### Domains of Feasibility and Measures

Bowen et al. (2009) suggested an evidence-based framework for feasibility studies consisting of eight domains: demand, practicality, acceptability, implementation fidelity, adaptation, expansion, integration, and limited efficacy testing. In the present study, the first four domains were used to evaluate the feasibility of the novice physical activity instructors’ leadership training program. The definition of each domain, the types of data, and the measures used to assess the domain are presented in Table 1. All the data were collected in Finnish.

**Table 1:** The definitions, types of data, and measures of the examined feasibility domains of Bowen et al.’s (2009) evidence-based framework.

Domain	Definition	Types of data	Measures
Demand	The extent to which a program is likely to be used or how much demand is likely to exist (Bowen et al., 2009)	The number of expressions of interest for the training	Emailed expressions of interest to participate
	The extent to which there is a demonstrated need for the program in the community (Shields et al., 2018)	The number of applicants waitlisted	
		Novice instructors’ perceptions of the need for the training program	Researcher’s log
			Application videos

		Expressions of public interest and demand for training novice physical activity instructors	Personal contacts from different stakeholders
Practicality	The extent to which the program can be successfully delivered to intended participants using existing means, resources, and circumstances (Bowen et al., 2009)	Evaluation of program delivery and the existing means, resources, and circumstances	Training intervention feedback form (Renko et al., 2020)
			Researcher’s log
	The extent that there are any adverse effects on participants (Shields et al., 2018)	Number and content of adverse events	Conversations retrieved from the video recordings
			Researcher’s log
			Novice instructors’ lesson plans
			Semi-structured focus group interview
Acceptability	The program recipients’ (i.e., novice instructors’) and program deliverers’ (i.e., trainers’) anticipated and experienced cognitive and emotional responses to the intervention, measured prior to (prospective acceptability), during (concurrent acceptability) or after (retrospective acceptability) the intervention (Bowen et al., 2009; Sekhon et al., 2017)	Novice instructors’ and trainers’ expectations and experiences of the program	Open-ended question about expectations
			Application videos
			Conversations retrieved from the video recordings
			Training intervention feedback form (Renko et al., 2020)
			Semi-structured focus group interview

		Novice instructors’ perceptions of autonomy support and relatedness in the training	Novice instructors’ lesson plans
			Researcher’s log
			Acceptance subscale of the need for relatedness scale (Richer and Vallerand, 1998)
			Instructors’ perceptions of autonomy support in the training intervention (Quested and Duda, 2011)
			Researcher’s log
Implementation fidelity	The degree to which program is implemented as intended by the program developers (Dane and Schneider, 1998; Carroll et al., 2007; Breitenstein et al., 2010)	See Table 2	See Table 2

The implementation fidelity of the novice physical instructors’ leadership training program was evaluated in terms of four dimensions (adherence, dose, quality of delivery, and program differentiation) to provide a comprehensive picture of the program integrity (Dane and Schneider, 1998). See Table 2 for definitions, types of data, and measures of each dimension of implementation fidelity.

**Table 2:** The definitions, type of data, and measures of the examined implementation fidelity dimensions.

Dimension	Definition	Type of data	Measures
Adherence	The extent to which specified program is delivered as originally designed by the program developers and described in the program manual (Dusenbury et al., 2003; Mihalic, 2004)	Comparison of the implemented and planned programs	Protocol (Toivonen et al., 2021)
			Researcher’s log
			Trainers’ lesson plans
Dose or exposure	The level at which the intervention is delivered to participants. It consists of number, amount frequency, and duration of the meetings (Dane and Schneider, 1998; Ibrahim and Sidani, 2016)	Comparison of the implemented and the planned number of meetings, length of each meeting, frequency of the meetings, and the total length of the training	Protocol (Toivonen et al., 2021)
			Researcher’s log
Quality of implementation	The trainers’ competence, such as trainers’ skills, attitudes, knowledge, belief in the training, preparedness, and motivation to deliver the program (Mihalic, 2004; Ibrahim and Sidani, 2016)	Description of the trainers’ education	
		Trainers’ perceptions of the training	Researcher’s log
			Conversations retrieved from the video recordings
Program differentiation	The identification of unique features of the components of the program that distinguish it from other programs (Dusenbury et al., 2003)	Comparing the core components of the program to other training programs found in the literature	Protocol (Toivonen et al., 2021)
			Researcher’s log
	An analytic process to determine the degree to which these core components that distinguish one program from another are present or absent (Century et al., 2010)		
		The degree of the presence of the distinguished core components	
			Protocol (Toivonen et al., 2021)
			Researcher’s log

Novice instructors submitted *application videos* during the recruitment phase. In the application videos, they reported their age, gender, education, occupation, physical activity background, favorite sports, instructing experience, instructing training, and motivation to participate in the training program and work with young people. The application videos were used in the evaluation of the demand and the trainers’ acceptability of the leadership training program.

*Novice instructors’ expectations* toward the training program were assessed qualitatively prior to the training with a written answer to an open-ended question (What do you expect from the training?). These were used to evaluate the novice instructors’ acceptability of the leadership training program.

*Training intervention feedback form* (adapted from Renko et al., 2020) was used to qualitatively and quantitatively assess novice instructors’ perceptions of the acceptability of the leadership training program and the trainers’ expertise. The feedback form consisted of five open-ended questions (e.g., “How could the training intervention be improved?”) and 14 statements (e.g., “I was satisfied with the training intervention,” “I understand the TPSR model well,” and “The training intervention included appropriate amount of theory”) rated on a 5-point Likert scale (1=strongly disagree and 5=strongly agree). The novice instructors filled out the feedback form after the training program.

*Novice instructors’ and trainers’ lesson plans* of the practice teaching lessons and model lessons were used to qualitatively assess the practicality and the adherence of the leadership training program. The lesson plan template included background information (i.e., date and place of instruction, number of students, name of the instructors, and topic and life skills of the lesson) and the plan for the lesson (i.e., physical activity and life skill goals, lesson content divided into awareness talk, physical activity time and group meeting/reflection time, as well as time spent on each activity and other comments). The novice instructor pairs filled out the lesson plan template prior to each practice teaching lesson and trainers prior to the model lessons.

A 30-min semi-structured *focus group interview* was organized four months after the training program. The focus group interview qualitatively assessed the novice instructors’ experiences and perceptions of the practicality and acceptability of the leadership training program (e.g., “What did you like best about the training program?” or “What would you have liked to have more in the training program?”). The focus group interview was video recorded.

*A researcher’s log* was used prior to, during, and after the leadership training program to qualitatively assess the trainers’ perceptions of all domains of the feasibility of the leadership training intervention. The researcher’s log included field notes, researchers’ perceptions of the training, and a track of attendance, components delivered, and time used. In addition, adverse events were monitored by the trainers and addressed in the researcher’s log.

The model lessons, practice teaching lessons, and following conversations and reflections were video recorded. *Conversations* were then retrieved from the video recordings, transcribed, and used to qualitatively assess the novice instructors’ and trainers’ perceptions of the acceptability of the leadership training program. The conversations were part of the planned reflection time of the lessons.

*Acceptance subscale of the need for relatedness scale* (Richer and Vallerand, 1998) was a 5-item scale used to quantitatively assess the novice instructors’ perceptions of relatedness in the leadership training intervention and to evaluate the acceptability of the training program. Minor adjustments in wording were made to enhance the items’ relevance to the leadership training intervention (“In this training program, I felt…” followed by items, such as “supported,” “valued,” and “safe”). Ratings were based on a 5-point Likert scale (1=strongly disagree and 5=strongly agree). The novice instructors filled out the scale at the end of the leadership training intervention. The scale has demonstrated adequate reliability in the previous research (Renko et al., 2020).

*The perceived autonomy support questionnaire* (Reinboth et al., 2004; Quested and Duda, 2011) was a 7-item questionnaire that quantitatively assessed the degree to which the novice instructors perceived trainers as supporting their autonomy. The questionnaire was used to evaluate the novice instructors’ acceptability of the leadership training program. Minor adjustments in wording were made to enhance the items’ relevance to the training intervention (e.g., “Trainers provided me with choices and options.”). Ratings were based on a 7-point Likert scale (1=strongly disagree and 7=strongly agree). The novice instructors filled out the questionnaire at the end of the leadership training intervention. Measure of perceived autonomy support has demonstrated construct validity and internal consistency in the previous studies (Reinboth et al., 2004; Quested and Duda, 2011).

### Data Analysis

Descriptive statistics (means and standard deviations) were used to analyze the quantitative data and assess practicality, acceptability, and implementation domains of feasibility of the training program.

Deductive and inductive content analyses were used to analyze the qualitative data and assess the feasibility of the training program (Lincoln and Guba, 1985; Flick, 2014; Patton, 2015). Four (i.e., demand, practicality, acceptability, and implementation fidelity) domains of feasibility evaluation (Bowen et al., 2009) and four dimensions (i.e., adherence, dose, quality of delivery, and program differentiation) of implementation fidelity (Dane and Schneider, 1998) were used as categories in the deductive content analysis, which was used to analyze the presence, absence, and content of these categories. Data were organized on a timeline, and changes over time were studied inductively. For example, changes in the openness of the participants are described based on this analysis.

The first author was responsible for all the analysis, but she was in constant dialogue with the co-authors who carefully followed up on the whole analysis process as suggested by Elo et al. (2014). The researchers critically assessed their own actions throughout the training program and during the data analysis to prevent the researcher bias and ensure the trustworthiness of the collected data and the content analysis. The credibility of the analysis was confirmed by careful selection of the most appropriate methods of data collection and checking for the representativeness of the data.

## Results

The feasibility of the novice physical activity instructors’ leadership training program was evaluated across four domains: demand, practicality, acceptability, and implementation fidelity (Bowen et al., 2009).

### Demand

Fifty-six applicants expressed an interest in participating in the study, the majority within the first week of recruitment. The extent of the leadership training program (20 h), the timing of the training (summer), other inclusion criteria, and typically low response rate for messages sent through the message boards and mailing lists were expected to significantly limit the number of applicants. However, the demand still clearly exceeded what the research team could organize within the timeframe and existing resources. Eight novice instructors were selected, and nine were waitlisted.

Demand for the training program was also demonstrated by the lack of other leadership training programs available for the novice instructors. The TPSR model had never been used for novice instructors, and there were no other programs teaching life skills for novice instructors in the city or the entire country. The city hires instructors for afterschool physical activity clubs by collaborating with sport clubs. Sport clubs recruit their young athletes to run the clubs without any training or experience, which lead to them merely providing equipment and no instruction. The participants of the afterschool physical activity clubs nor the novice instructors learn any leadership skills.

The demand for the program became even more apparent during the leadership training program, when the novice instructors brought up that they had never before consciously practiced self-expression, evaluated themselves and others after a physical activity session, or intentionally practiced leadership skills. Laura (female, 18 years) stated,

*We are expected to make really big decisions regarding our lives, our future. It’s a lot of responsibility. And we are just expected to know how to make these decisions. How is it possible that this is the first time I’m hearing about these [life] skills and actually practicing them? Like, these are so important skills. These should have been taught to us a long time ago*.

The novice instructors were not familiar with shared leadership either. Their previous experiences included vertical leadership with the formal leader being hierarchically placed above the followers. Therefore, there was also a demand for learning shared leadership.

Additionally, after the intervention, different organizations heard about the training program. Two cities, several sport associations, and the Finnish Olympic Committee expressed their interest in the program. Consequently, the program has already been disseminated in Finland to novice coaches with immigrant backgrounds as part of the Erasmus+ Sport Peer education, Leadership, Action, Youth! (PLAY) project funded by the European Union and to experienced coaches and educators who train novice physical activity instructors as part of the Hood Coach project funded by the city of Helsinki. These results indicate a demand for leadership training among young novice instructors.

### Practicality

#### Resources and Circumstances

The host university provided the required physical activity equipment and spacious facilities for the training. During the third practice teaching lessons, the sport teams provided the required equipment. Without these resources, the program could not have been delivered according to the training manual.

The first author also created a Web site in Finnish, which was used during the leadership training program and beyond. It included all the relevant materials and information concerning the leadership training program and the study. The novice instructors were able to access it at any time from any device. The Web site proved useful during the training, especially when the novice instructors were planning their practice teaching lessons, but in other times, the novice instructors were not utilizing it. At the beginning, the novice instructors also reported some challenges accessing the password-protected Web site.

The novice instructors were particularly pleased with the snacks. Four of them mentioned them in the anonymous training feedback form, and it was the first thing they mentioned during the focus group interview four months after the training when asked what they remember about the training.

The group size of eight was optimal for the leadership training program with the existing resources. It allowed sufficient individual contribution from each novice instructor, individual feedback, and a large enough group to organize the first practice teaching lessons.

#### Timing and Duration

The participants considered the timing of the training convenient; however, during the recruitment, a few applicants indicated they could not participate due to the timing. The trainers considered timing of the training optimal for the target group as it was organized before the new school year started.

The participants considered the duration of the training appropriate. Although they may have liked more practical training, they acknowledged that participation would have been more difficult if the training was extended. Jesse (male, 22 years) summed it up, *“If the training was longer, I couldn’t have made it. Now, I was able to quit my summer job a couple of weeks earlier and made it.”* The trainers considered 20 h as appropriate, sufficient, and necessary duration for the novice instructors’ leadership training program. More meetings or longer training days would have required more resources. Less meetings or shorter training days would have made it impossible to cover all the planned content.

The trainers and the novice instructors were pleased with the timing of the meetings. Leo (male, 18 years) stated, *“It’s good to have to wake up early because school starts soon. And we are done early so plenty of time to still relax.”* Seven meetings were considered appropriate, and three hours would have been the optimal length of a meeting, because in the meetings that lasted three and half hours, the novice instructors started yawning and losing their focus even though the content was very interactive. The meetings were organized frequently enough for the participants and the trainers to maintain their focus on the training but not too frequent to become consumed by the training. In addition, the total length of time over which the intervention was given (five weeks) was perceived suitable by the participants and the trainers.

#### Adverse Events

No major adverse events were reported, which indicates practicality. A few minor adverse events were reported and observed. For example, when only four female volunteer athletes showed up for the second practice teaching lessons instead of 10, the novice instructors were surprised and became worried and anxious because they had to modify their lesson plans on the spot. During the debrief after the second practice teaching lessons, Laura talked about this challenge, *“There were so few of them that time was not spent on like dividing teams or things like that. So, we had a little more time than we had thought of*.” Her partner Anna (female, 19 years) continued, *“There were a few games that we had not planned to do either but then decided to take them.”*

Some physical fatigue was reported by the novice instructors during and after the third and the fourth meeting. At the end of the fourth meeting, Tom (male, 18 years) stated, *“I can feel that it’s been quite a lot of practice for my legs in the past few days and I still need to bike home.”* Additionally, despite having snacks available, some novice instructors reported being hungry at the end of the third and the fourth meetings that had included plenty of physical activity.

### Acceptability

#### Novice Instructors

The novice instructors perceived the training program highly acceptable. The descriptive statistics of the novice instructors’ evaluations of the acceptability of the training program can be found in Table 3.

**Table 3:** The descriptive statistics of the novice instructors’ evaluations of the acceptability of the training program.

What do you think of the training?	*M*	*SD*
Training was as I expected	4.14	0.69
Training was demanding	3.29	0.95
Participating the training was fun	4.57	0.53
Amount of theory was appropriate	4.43	0.53
Training was useful	4.71	0.49
I was satisfied with the training	4.71	0.49
Amount of practical application was appropriate	4.29	1.11
Trainers had expertise	4.86	0.38
I would recommend the training to others	4.57	0.53

*5 = strongly agree to 1 = strongly disagree*.

Prior to the training, the novice instructors gave a written answer to an open-ended question about their expectations toward the training program. Their expectations included developing their leadership skills, spending quality time with likeminded people, and receiving comprehensive and useful training. In the anonymous training feedback form, the novice instructors considered the training being as they had expected. Some novice instructors also became close friends with each other outside of the leadership training.

The attendance rate in the leadership training program was 100 percent. The novice instructors accepted the training program and its core components (theory, activity, experiential learning, evaluation, and leadership), and engaged in all the content. None of the novice instructors had heard about the TPSR model before the leadership training program or had any experience with it. Anna also brought this up in a conversation during the training,

*It [the TPSR model] was a brand new thing. It was not familiar to me at all. Even though I have participated in organized sports and been in school for so many years, I have never come across anything like this*.

When the novice instructors applied for the program, they knew the extent of the training and that they would be trained to lead afterschool physical activity clubs. However, it became apparent that the novice instructors had never deliberately practiced or even considered life skills and were not expecting to learn a model or have a theory being taught to them and to base their instruction on. During the focus group interview, some novice instructors mentioned how having a theory component had surprised them. Tom explained,

*At first I was like, this is a physical activity program. So, I did not think that there needed some skills to be transferred and talk about deep stuff. And that kind of hit me a little like what, what is this?*

Jesse was also surprised by the extent of the theory, *“Theory that we had …it was at first quite, well, like it was more than expected or what I had personally thought of.”* Despite the surprises, the novice instructors considered the training to have a good amount of theory. Jesse continued, *“I do not mean there should have been less theory but maybe more practice…if there was time for it.”* In the anonymous training feedback, some of the novice instructors specifically thanked for the systematic implementation of the TPSR model throughout the program, *“I liked it that the same things were repeated often and from many angles”* and *“I liked it that the same things were gone through first in theory and then in practice.”*

At the beginning of the training, the self-expression activities were challenging for the novice instructors and they did not feel safe enough for personal risk taking. For example, when the trainer explained the activity, *“Its berry season now. Think about what berry you would be and why. [break] Why don’t we start with Jesse first.”* Jesse was surprised and struggled to express himself, *“Oh…well…berry…now this was such a random question at this point that [silence, thinking]…for example [silence].”* The trainer helped him, “*What first comes to your mind?”* and Jesse answered, *“For example gooseberry.”* Jesse was looking at the trainer and expecting her to call the next person’s name. Everybody was quiet and waiting for Jesse to continue. Soon he realized it was still his turn, *“Oh, do I need to? I need to give reasons. Right…umm…it’s big and the bush has a lot of spikes.”* When it came to Tom’s turn, he was also struggling to answer, *“[nervous laugh] Em…hmm…well…em.”* Jesse said sarcastically, “*A lot of time to think.”* Tom smiled and responded, “*It’s a hard task. Hmm. Well, probably strawberry because it is, hmm, big and good.”*

Despite the challenging start, over the course of the training the self-expression and team-building activities worked well in getting the novice instructors to interact, feel safe in the group, take initiative, express themselves, and enjoy the training. During the third meeting, which was in a gymnasium instead of a classroom, the novice instructors opened up and from that point on became gradually more engaged in all the activities. For example, during the first practice teaching lessons in a story activity that Anna was leading with Laura, she struggled to continue a story, but the other instructors actively helped her. Anna stated, *“Once upon a time there was a little boy who was skinny who had [laughing]…no…how…I don’t remember.”* Jesse and Aaron (male, 18 years) simultaneously helped Anna, *“Was very skinny.”* Anna continued repeating the story, *“But who had strawberries that smelled like moss. Once…”* and passed the ball to Heidi (female, 18 years). Laura who was leading the activity stated, *“Now, can we still get it right?”* Heidi tried to repeat the story, *“Once upon a time there was a very little boy…no…[paused]”* Many of the instructors supported Heidi and simultaneously said, *“Yes!”* Jesse corrected them, *“No, a skinny little boy.”* Everybody laughed. Leo stepped in, *“No it did not go like that. [Emma repeated simultaneously with Leo] Once upon a time, there was a little boy, who was very skinny. [Leo continued alone] It was [paused and was thinking]”* Ville added, *“We can check this from the video!”* and everybody laughed.

All the novice instructors completed the three practice teaching lessons and were enthusiastic about them. The novice instructors considered the training having a good amount of practical training, “*We were allowed to apply a lot in practice.”* However, in the focus group interview, Jesse was also hoping for more practice with the peers before the volunteers, “*I would have liked to have more, for example, practice with the own group so that I would have gained some more confidence before leading the group of strangers.”* For the practice teaching lessons, the trainers had assigned the pairs based on age, gender, sport background, instructing experience, and personality to generate as heterogeneous pairs as possible of the same gender. However, some of the novice instructors would have liked to work more with different partners and choose their partners for the practice teaching lessons. Two of the pairs were functioning very well from the beginning, but the other two pairs had more challenges with their cooperation. The same pairs were kept for all three practice teaching lessons to give them a chance to develop better communication and teamwork.

All the novice instructors completed all required self-evaluations and reflected on their instructing performance after each practice teaching lesson. The novice instructors also gave each other good, relevant, and positive feedback. For example, Jesse gave feedback to Tom and Aaron after their first practice teaching lesson,

*Seriously boys, a great performance! I wouldn’t have personally even known how to start to do what you just did. It requires letting go, which came naturally from both of you. Dance is that kind of, at least for me, it immediately makes me feel uncomfortable. Like, now I should dance and move smoothly in front of others. Boys did very well the whole lesson and it was a really professional lesson. It was fun!*

The novice instructors reported that participating in the training was somewhat demanding. One novice instructor also referred to the burden in the anonymous training feedback when asked what he/she did not like about the training, *“Too big workload and too high expectations of it.”* Despite being somewhat demanding, the training was perceived as fun and useful by all the instructors and they were satisfied with the training as one of the instructors expressed in the anonymous feedback, *“The training was clear and efficient. Things that were agreed upon were taken care of.”*

All the novice instructors would recommend the training to others and rated the training overall as very good (*M* = 4.50, on a 5-point scale, *SD* = 0.53). In the training feedback questionnaire, Aaron further brought up his satisfaction with the training, *“Very comprehensive training from which a novice instructor gets a good foundation as long as he/she has the courage to participate.”* as did Jesse, *“The training was good. I believe it will be useful for me in the future.”*

The participants perceived their basic psychological needs of relatedness (*M* = 4.73 on a 5-point scale, *SD* = 0.51) and autonomy (*M* = 6.54 on a 7-point scale, *SD* = 0.69) being strongly supported during the training program. This demonstrates that the trainers shared leadership and were able to engage the novice instructors. According to the anonymous feedback after the training, the novice instructors also considered the trainers’ expertise high, *“The trainers in my opinion were really good and tried to help us.”*

#### Trainers

Based on the application videos, the trainers expected the chosen novice instructors to be quite outgoing. Therefore, it came as a surprise how reserved the group and shy the participants were at first. Despite the hesitations in the beginning, the leadership training went better than the trainers had expected. The trainers were especially pleased with all the activities, the model lessons, and the practice teaching lessons. The trainers enjoyed the training and, on several occasions, expressed their feelings to the novice instructors during the training, *“I have really enjoyed training you. This is a great group and I have a really good feeling about tomorrow.”*

The trainers had invested time and effort into gaining an in-depth understanding of the TPSR model in theory and in practice prior to the training. This preparation is described later in results. Despite the extensive preparation, the training was demanding for the trainers. It was long and intense, and required the trainers to focus on the implementation of the TPSR philosophy throughout each meeting. However, the challenge also made the program attractive to the trainers, as it required them to pay careful attention to their own behavior and reflect during and after each meeting.

The only thing the trainers were not fully satisfied with were the observations of the sport practices (i.e., sixth meetings). It was beneficial for the novice instructors to contact the coaches, organize the observations, see the coaches and athletes in action, and provide feedback to the coaches. However, the novice instructors and the trainers struggled to maintain their focus for the entire 60 min, as some of the practices did not have much variability in the content or active coaching. In addition, all the observed coaches were male.

### Implementation Fidelity

The implementation fidelity of the novice physical activity instructors’ leadership training program and its core components (i.e., theory, activity, experiential learning, evaluation, and leadership) were evaluated in terms of four dimensions: adherence, dose or exposure, quality of delivery, and program differentiation (Dane and Schneider, 1998).

#### Adherence

The leadership training program and all its core components were delivered as originally designed by the program developers and described in the program manual. One group division activity was changed during the training to a more psychologically safe one to better fit the needs of the group.

#### Dose or Exposure

The originally prescribed level of the leadership training program was delivered to the novice instructors. The overall length of the training program was 20 h and duration five weeks. The leadership training program consisted of seven meetings ranging from 135 to 210 min. The first three meetings were arranged one day apart during week one, the next two meetings were organized during the first two days of week two, the sixth meetings were completed in pairs during week three, and the last meetings were organized in pairs during week four and five.

The theory component consisted of approximately 90 min of lecturing and 30 min of discussing the model during the second meeting. Approximately, three hours were spent reviewing the content of the theory component throughout the rest of the training. The activity component consisted of a group guidelines activity, five self-expression activities, eight team-building activities, and seven different ways to divide a group, totaling up to approximately two hours. Each novice instructor participated the two 60-min model lessons led by the trainers during the third meeting and three 30-min practice teaching lessons (i.e., first practice teaching lessons) led by their peer novice instructors during the fourth meeting, constituting the experiential learning component. The evaluation component consisted of the novice instructors’ evaluations of themselves after each practice teaching lesson (fourth, fifth, and seventh meetings), their peers after the first and second practice teaching lessons (fourth and fifth meeting), the trainers after the model lessons (third meeting), and the sport coaches after the observation of a sport practice (sixth meetings). In addition, it included the trainers’ evaluations of the novice instructors after each practice teaching lesson (fourth, fifth, and seventh meetings), the sport coaches after the observation of sport practices (sixth meetings), and themselves and the other trainer after each meeting. The leadership component consisted of two 30-min practice teaching lessons (fourth and fifth meeting) for each pair organized one day apart during week two and a 60-min practice teaching lesson (seventh meetings) for each pair during weeks four and five. Additionally, different leadership and responsibility tasks were given to the participants throughout the training program demonstrating shared leadership.

#### Quality of Delivery

To ensure quality of the theory component, prior to the training, the trainers acquired an in-depth understanding of the TPSR model and used the principles of the model in their teaching and coaching. Both trainers had discussed with and received guidance from Dr. Don Hellison, the creator of the TPSR model. Both trainers also participated in a small group TPSR training led by the third author. The first author received additional one-on-one training by the third author, including observation and feedback on TPSR implementation and training on how to evaluate TPSR programs for implementation fidelity. The first author also met other members of the TPSR community of practice (i.e., TPSR Alliance) and observed their programs’ use of the principles of the TPSR model.

The trainers had used the group guidelines and most of the self-expression, team-building, and random group division activities of the program with several different groups and sport teams in the past. Based on their previous positive experiences, the trainers believed that the activity component increases the individuals’ self-awareness and the group’s cohesion and were motivated to organize the activities. The trainers also participated in the activities, which further improved the trainer-novice instructor relationships.

Both trainers had extensive experience in instructing physical activity and providing feedback to groups and individuals including novice instructors. They considered the evaluation component as an important tool to influence the novice instructors’ future instructing behavior, self-efficacy, and skills to receive and provide feedback. The trainers gave plenty of positive feedback to the novice instructors,

*For many of you this was the first time that you led a whole physical activity or any practice. It went great. Awesome. You used so many of these more advanced, challenging teaching strategies that not necessarily experienced, trained, professional teachers and coaches know how to use*.

They also gave constructive feedback when it was needed. For example, when a novice instructor had stood with her arms crossed for a long time during a practice teaching lesson, the trainer brought it up during the following debrief by asking, *“What feeling do you get if I’m like this [crosses her arms]? Or likewise if I’m holding my hands in my pockets?”* Tom responded, *“That kind of like you are not interested”* and Jesse added, *“You are more difficult to approach.”* The trainer continued, *“Exactly. So, pay attention to your body language because you signal a lot with it, in addition to the things that you say.”*

To ensure the quality of the experiential learning component and the leadership component, the trainers carefully followed the training manual. Both trainers had extensive leadership experience, and they had acquired TPSR-based philosophy that emphasized shared leadership, which was the focus of the experiential learning component and the leadership component. Shared leadership was embedded throughout the training program, and the novice instructors’ leadership and responsibility were progressively increased. This was also made concretely known and visible to the novice instructors. Once the trainers had managed to establish a good relationship with the instructors, they gave them a vast variety of individual managerial and leadership tasks. The trainers had also chosen activities throughout the training that supported and required autonomy from the instructors. During the model lessons, the trainers gave each instructor opportunities to take a lead and make decisions individually and as a group. Through following discussions, the novice instructors were asked to identify how the trainers supported the novice instructors’ autonomy, what kind of leadership tasks they were given, and how they could lead physical activity groups accordingly. During the practice teaching lessons, the instructors were given autonomy to choose the content of the lessons (i.e., physical activity and life skills) and lead the group with their partner. The trainers were only observing and providing feedback afterward. Shared leadership was also emphasized in the discussions, and the novice instructors were reflecting and evaluating how they managed to share leadership. These results are presented elsewhere.

The trainers were experienced, motivated, and competent to deliver the training program and all its core components. These trainer qualities were vital in achieving high fidelity of implementation of the training program. Furthermore, having two trainers significantly improved the quality of delivery. For example, one trainer could observe the lesson, while the other one was leading it. Also, if one trainer was forgetting something, the other one stepped in, or if one trainer was asking an unclear question, the other one clarified. The trainers also engaged in critical self-reflection and discussed implementation during and after the meetings.

#### Program Differentiation

The novice physical activity instructors’ leadership training program was based on the TPSR model and covered the model both in theory and in practice. This differentiated the program from a typical physical activity leadership training program, which includes either lectures about a theory without practical implementation or practical training without a theoretical basis.

The uniqueness of the novice physical activity instructors’ leadership training program compared to other published TPSR-based physical activity instructors training programs was strongly related to the extensiveness of the core components of the training. Unlike other published TPSR-based physical activity instructors training programs, this leadership training program included an extensive amount of self-expression, team-building, and group division activities. The experiential learning component of the current leadership training program had a unique focus on experiencing the model in practice teaching lessons as a peer participant. Also, live observations of sport practices were included in this leadership training program. In TPSR-based training programs, participants are typically given leadership roles and empowered in the training process. This was also the case in the current study. Additionally, all the novice instructors led practice teaching lessons to three very different target groups (i.e., peers, volunteer athletes, and a sport team or group), whereas TPSR-based training programs typically offer real-life leadership opportunities only with a certain target group or only to some of the participants.

## Discussion

Results indicate that the leadership training program for novice physical activity instructors was feasible in the current context with the available resources. There was a demand for the training program, and the training was practical and highly acceptable by the novice instructors and the trainers. The leadership training program was also implemented with high fidelity. Therefore, it has a high probability of efficacy and it can be deemed acceptable for further evaluation.

### Demand

Previous research on leadership development has focused almost exclusively on adult leadership (Karagianni and Montgomery, 2018). However, adult leadership models do not consider adolescents’ and young adults’ unique developmental needs (Linden and Fertman, 1998). Therefore, there is a need for leadership development in young people (Gould et al., 2006; Martinek and Hellison, 2009).

The demand for young adults’ leadership training was also demonstrated in the current study. In a matter of weeks, 56 interested participants were reached, exceeding the intake. Due to limited resources, many motivated novice instructors could not be trained. During the leadership training program, the novice instructors continuously expressed their lack of experience in responsibility and leadership skills and claimed that they have not been given opportunities to learn these skills. Perhaps, the skills are practiced at school, but the students are not made aware that they are practicing the skills. Teachers might not be telling the students why it is important, for example, to evaluate oneself, how to do it, and how to transfer the skill to other settings but instead just expects them to learn by themselves. The lack of programs that teach leadership skills to novice instructors means that most afterschool physical activity clubs are run by young inexperienced and untrained instructors. Therefore, in order to improve the quality of the instruction and the programs, and to promote shared leadership, there is a need for further leadership training that deliberately teaches responsibility and leadership skills and the transfer of the skills to novice instructors. The demand has also been reflected in the nationwide interest toward the leadership training program after the training intervention was first presented to the public.

### Practicality

The training program was successfully delivered to intended participants using existing means, resources, and circumstances. The host university and sport clubs assisted with the equipment. The timing and duration of the training and the meetings were appropriate for the target group and in line with previous research on TPSR-based leadership training programs, which have shown promising learning outcomes for teachers and coaches trained in a 20-h TPSR training (Escartí et al., 2013) and in intensive TPSR workshops over the course of a week (Wright et al., 2016, 2018). Extending the duration of the training program would have required more resources and could have reduced the number of available participants. The group was optimal size. Larger number of participants would have required a longer training period and more resources and would have reduced the instructors’ individual contribution, which all could have influenced the outcomes of the training program. In previous studies, instructor trainings have been typically organized for one to eight teachers or coaches (e.g., Escartí et al., 2010, 2018; Beaudoin, 2012; Hemphill et al., 2015; Lee and Choi, 2015; Wright et al., 2016). When larger groups of instructors are trained, the number of trainers increases and, for example, concurrent sessions are organized in order to divide the group into smaller groups and to maximize interaction (Wright et al., 2018).

Lack of major adverse events also indicated practicality. Even having less volunteer athletes than expected provided a lesson of how the instructors need to be flexible and able to adjust their lesson plan.

### Acceptability

The novice instructors considered the training program somewhat demanding but fun and useful. They were satisfied with the training and would recommend it to others. High satisfaction was found also in other TPSR-based leadership training programs (e.g., Wright et al., 2018). Although the novice instructors felt that their autonomy and relatedness were highly supported during the training program, they also would have liked to have the opportunity to choose their partners for the practice teaching lessons.

All the novice instructors participated in the training program and engaged in all its content. Although none of them had heard about the TPSR model before the training program, they received it well. They especially liked that the model was systematically brought up and followed throughout the training program. Self-expression and evaluation were challenging for them at the beginning of the training. However, once the group had become safe enough for personal risk taking, they opened up more. All the novice instructors completed the three practice teaching lessons and were enthusiastic about them. All the novice instructors also completed all required self-evaluations and reflected on their instructing performance after each practice teaching lesson. Their feedback to each other was appropriate, accurate, and helpful. The trainers were perceived competent by the novice instructors.

The trainers were satisfied with the novice physical activity instructors’ leadership training program. At first, the novice instructors were shy and reserved but in the end the training program surpassed the trainers’ expectations. The trainers had prepared well and although the training was intense and demanding, the trainers enjoyed it. The only thing they were not fully satisfied with was the observations of the sport practices (i.e., sixth meetings) because some of the practices were too long with not much variability in content. Therefore, in the future, the observations could be replaced with watching video recordings of the trainers instructing the model lessons or the novice instructors instructing the practice teaching lessons. Alternatively, video recordings of female and male and novice and experienced coaches leading teams of different sports and at different competitive levels could be used. Also, if available, videos of TPSR-based programs could be observed as in the teacher education of Escartí et al. (2018).

### Implementation Fidelity

The novice physical activity instructors’ leadership training program and its core components were delivered to novice instructors as originally designed and prescribed by the program developers in the program manual. The trainers of the leadership training program had acquired an in-depth understanding of the TPSR model in theory and in practice, which is essential for a quality delivery of the TPSR. The training program cannot be executed without the experienced, motivated, and competent trainers. The trainers also acquired the mindset of TPSR as “a way of being” for the program leaders, not just a way of teaching (Hellison, 2011).

In their systematic review of PYD in sport, Holt et al. (2017) found that theoretical models and conceptual frameworks were used sparingly in PYD programs. Therefore, it is safe to assume that PYD-based leadership training programs are rarely based on theory and the presence of the theory component distinguishes the current novice physical activity instructors’ leadership training program from most other PYD-based leadership training programs.

The creation of positive relationships between the leaders and the students and among the students is at the heart of the TPSR model (Hellison, 2011). Therefore, most TPSR-based instructor training programs include some team-building games (Wright et al., 2018; Alcalá et al., 2019). However, tasks to randomly divide groups or self-expression activities have not been mentioned in the TPSR instructor training literature, although Gordon et al. (2016) showed that the TPSR model aligned strongly with the social emotional learning framework, which is a context in which these activities are commonly used (Lintunen and Gould, 2014). These kinds of activities were not the focus of the leadership training program, but they were crucial in creating positive relationships and a psychologically safe learning environment.

The experiential learning component was especially important for the novice instructors because they had never experienced shared leadership or the TPSR model in practice before. The model lessons and first practice teaching lessons provided experiences of how it feels to participate in TPSR-based lessons and demonstrated how to embed shared leadership and the TPSR model in physical activity lessons. Wright et al. (2018) also organized demonstration lessons to coaches in their training program, but the focus was on the latter purpose. Other TPSR-based instructor training programs have not reported providing any form of demonstration lessons for either purpose.

The current leadership training program was built around the concept of shared leadership. Leadership was gradually shared among the novice instructors along with personal and social responsibility. This was done transparently so that the novice instructors knew why the trainers did what they did. In other training programs where people were being trained to use TPSR as a teaching tool, shared leadership was also applied (Wright et al., 2016, 2017, 2018). The people being trained were given leadership roles and empowered in the training process. They shared the leadership roles as a group, provided leadership to their peers who were not on the leadership team, and implemented leadership strategies among youth participants in their program, in a way repeating the cycle. However, typically in other TPSR-based instructor training programs, only some of the coaches are invited to lead a single session (Wright et al., 2018) or all lead but only one familiar group, for example, their students (Hemphill et al., 2015; Lee and Choi, 2015) or coaches (Jacobs et al., 2020). Therefore, the current study was unique because the novice instructors were given opportunities to lead three very different target groups (i.e., peers, volunteer athletes, and a sport team or group).

Reflection and evaluation are core components of the TPSR model (Hellison, 2011). However, apart from professional development programs (e.g., Hemphill et al., 2015), it is difficult to evaluate the extent of reflection and evaluation in TPSR-based instructor training programs. In the current leadership training program, the novice instructors were reflecting and evaluating their own and other instructors’ performance after each practice teaching lesson and throughout the program. Additionally, in some TPSR-based instructor training, video recordings containing examples of the implementation of the TPSR model in physical activity have been used (Hemphill et al., 2015; Lee and Choi, 2015; Escartí et al., 2018). However, live observations of sport practices have not been previously reported. The main purpose of the live observations was to provide the novice instructors with an example of a real-life coaching situation and make them reflect on how to implement the TPSR model to it.

## Limitations

The novice physical activity instructors’ leadership training program was designed for the Finnish context where the current study was conducted in Finnish. Therefore, the culture may dictate the quality of the training program because, for example, interpersonal communication that is clear, supportive, and respectful in the Finnish context may be confusing and disrespectful in other contexts.

Adaptation, expansion, and integration domains of an evidence-based framework for feasibility studies (Bowen et al., 2009) were not investigated in this study. Therefore, it is unknown to what extent the program is feasible when implemented to different populations or settings, by different trainers, or into ongoing community practice. However, as the novice physical activity instructors’ leadership training program proved feasible, the training program can be attempted with different populations, trainers, and circumstances. Preliminary experiences with the Erasmus+ Sport PLAY! project indicated that the program can be successfully organized for novice instructors with immigrant backgrounds.

Additionally, the limited efficacy testing to examine the extent to which the training program works in making positive changes to the novice instructors’ responsibility and instructing behaviors will be presented elsewhere. Bowen et al. (2009) stated that for an intervention to be worthy of testing efficacy, it must address the relevant questions within feasibility. They also emphasized that researchers need to choose the domains that best match the needs of the situation. Hence, we examined demand, practicality, acceptability, and implementation fidelity to determine the feasibility of the leadership training program (Bowen et al., 2009).

One concern that might be leveled at our recruitment strategy might be the issue of self-selection, which we acknowledge. However, the goal was to recruit people who were sufficiently motivated to participate in our program, so, by definition, our recruitment process would recruit motivated, interested applicants. This strategy has also been used in other leadership programs for recruiting adolescents and young adults (Karagianni and Montgomery, 2018).

### Contribution to the Field and Future Directions

The leadership training program is the first TPSR-based training program for young novice physical activity instructors in Finland. Previous training programs in Finland targeted experienced physical education teachers (Rantala and Heikinaro-Johansson, 2007; Romar et al., 2015).

To our knowledge, this is also the first feasibility evaluation of a TPSR-based physical activity instructors’ leadership training program. Currently, there is no guidance for conducting feasibility studies, although Craig et al. (2018) are in the process of creating one for public health interventions. This study contributes to the evaluation research of TPSR-based programs by using an evidence-based framework for feasibility studies (Bowen et al., 2009), which can be used in the future to evaluate ongoing or new TPSR-based programs and leadership trainings until further guidance is developed. Therefore, this study responded to Martinek and Hellison’s (2016) call to discover new ways to evaluate TPSR-based programs to confirm fidelity of the programs and to provide ideas that can be applied to other programs.

## Data Availability Statement

The raw data supporting the conclusions of this article will be made available by the authors, without undue reservation.

## Ethics Statement

The studies involving human participants were reviewed and approved by The Ethics Committee of the University of Jyväskylä (No. 29062015). The patients/participants provided their written informed consent to participate in this study. Written informed consent was obtained from the individual(s) for the publication of any potentially identifiable images or data included in this article.

## Author Contributions

All authors were involved in designing the study and procedures. H-MT and TL collected the data and drafted the manuscript. H-MT analyzed the data. All authors edited the manuscript, read, and approved the submitted version.

## Conflict of Interest

The authors declare that the research was conducted in the absence of any commercial or financial relationships that could be construed as a potential conflict of interest.

## Publisher’s Note

All claims expressed in this article are solely those of the authors and do not necessarily represent those of their affiliated organizations, or those of the publisher, the editors and the reviewers. Any product that may be evaluated in this article, or claim that may be made by its manufacturer, is not guaranteed or endorsed by the publisher.
